# Supplementary material for: Personality intervention affects emotional stability and extraversion similarly in older and younger adults
Source: Commun Psychol. 2025 Nov 25;3:171. doi: 10.1038/s44271-025-00350-2 (PMC12647695; doi:10.1038/s44271-025-00350-2)
Supplement: Supplementary file 2 — Supplementary material with additional study information and analyses [file 44271_2025_350_MOESM2_ESM.pdf]

Supplementary Material to
Personality Intervention Affects Emotional Stability and Extraversion Similarly in Older and Younger Adults

Gabriela Küchler[1], Kira S. A. Borgdorf[2,3], Corina Aguilar-Raab[2,4] Wiebke Bleidorn[5], Jenny Wagner[6], and *Cornelia Wrzus[1,7]

[1]Department of Psychological Aging Research, Psychological Institute, Heidelberg University, Germany

[2]Clinical Psychology, Interaction- and Psychotherapy Research, Faculty of Social Sciences, University of Mannheim, Mannheim, Germany

[3]Heidelberg University, Heidelberg, Germany

[4]Institute of Medical Psychology, University Hospital Heidelberg, Heidelberg, Germany

[5]Univerity Zurich, Zurich, Switzerland

[6]University Hamburg, Hamburg, Germany

[7]Network for Aging Research, Heidelberg University, Heidelberg, Germany

Author Note

Gabriela Küchler https://orcid.org/0000-0002-5528-4958

Kira Skirwitt Ann Borgdorf https://orcid.org/0000-0002-1621-0876

Corina Aguilar-Raab https://orcid.org/0000-0001-9956-7047

Wiebke Bleidorn https://orcid.org/0000-0003-3795-8143

Jenny Wagner https://orcid.org/0000-0001-7507-9620

Cornelia Wrzus https://orcid.org/0000-0002-6290-959X

*Correspondence author: Cornelia Wrzus, Psychological Institute, Department of Psychological Aging Research, Heidelberg University, Bergheimer Straße 20, 69112 Heidelberg, Germany. Email: wrzus@psychologie.uni-heidelberg.de

Supplementary Table S1

Detailed Overview of Each Session during the 8-week Socio-Emotional Functioning Intervention

Content	Exercises/Reflection
**Module 1: Stress and Stress Synthesis**	
**Content**	**Exercises/Reflection**
Introduction to the training:<br>Training framework;<br>Change model<br><br>*Psychoeducation on stress:*<br>Definition of stress and stressors;<br>Stress theory (Lazarus & Folkman, 1984);<br>Physiology of stress;<br>Stressor-response sequence;<br>Parasympathetic and sympathetic nervous system<br><br>*How to approach sustainable change*	*Audio "Value reflection":*<br>Reasons for participation<br><br>*Written self-reflection:*<br>Analysis of the current and the desired state in dealing with stress;<br>Analysis of resources and obstacles for change;<br>Commitment to change goal(s)<br><br>*Audio "Nourishing moment":*<br>Visualization of a nourishing daily life moment (creating feelings of safeness, calmness, contentment)<br><br>*Everyday tasks:*<br>Audio "Nourishing moment";<br>Differentiation of pleasant and unpleasant body sensations;<br>Getting to know the Buddy
**Module 2: Resilience and Resources**	
**Content**	**Exercises/Reflection**
Psychoeducation on resilience:<br>Definition;<br>Resilient (elastic) zone;<br>Resilience factors;<br>Perspectives on level of functioning<br><br>*Collection of immediate help strategies;*<br>Dealing with anomalies and uncertainties<br><br>*Psychoeducation resources:*<br>Characteristics;<br>Internal and external resources	*Arrival with audio "Nourishing moment" & dyadic/ group exchange*<br><br>*Audio "Bodyscan":*<br>Conscious perception of body parts<br><br>*"Resource Shower":*<br>Getting to know own resources and strengths through external assessment<br>Goal: Promoting self- and other-awareness, enhancing group solidarity<br><br>*Written self-reflection:*<br>Personal strengths and resources;<br>Use of "immediate stress remedies"<br><br>*Everyday tasks:*<br>Audio "Bodyscan";<br>Identification of everyday stressors, own resources and immediate stress remedies;<br>"Resource interview": reciprocal interview with Buddy on strengths and resources
**Module 3: Attention and Attention Regulation**	
**Content**	**Exercises/Reflection**
Psychoeducation on attention:<br>Definition of attention and its core characteristics;<br>Stability versus clarity of attention;	*Arrival with audio "Nourishing moment" & dyadic/ group exchange*<br><br>*Audio "Focus on the Breath" & written self-reflection:*

Content	Exercises/Reflection
Benefits and mechanisms of attention regulation<br><br>*Psychoeducation on needs and values:*<br>Definition and differentiation of needs and values;<br>Identification of own needs and values	Conscious awareness of the breath to train attention;<br>Reflection on experiences of difficulty/ease with this exercise<br><br>*Written self-reflection:*<br>Identification of needs and values<br><br>*Everyday tasks:*<br>Audio "Focus on the Breath";<br>"Values Interview": reciprocal interview with Buddy on needs and values and how they relate to their resources;<br>One mindful daily activity per day (with attention to bodily sensations, thoughts, and feelings)<br>Exchange with the buddy
Module 4: Emotions and Emotion-Regulation	
**Content**	**Exercises/Reflection**
Psychoeducation on emotions and emotion regulation:<br>Definition of emotions, emotion regulation, stimulus-response patterns;<br>Differentiation of affect, mood, and emotion;<br>The role of attention in emotion regulation;<br>Ability to distinguish and self-regulate<br><br>*Psychoeducation on self-care:*<br>Definition of self-care;<br>Self-compassionate attitude;<br>Friendly and critical self-verbalization;<br>Negative thought and emotion spirals;<br>Acceptance versus change	*Arrival with audio "Focus on the Breath" & dyadic/ group exchange*<br><br>*Audio "Open Monitoring":*<br>Conscious awareness of all present-moment sensations/experiences;<br>Sharpen the attention<br><br>*Written self-reflection:*<br>Reflection on focus versus distraction ;<br>Identification of habitual stimulus-response patterns<br><br>*Exercise "Untangling a knot"*<br>Perceiving thoughts and body sensations with a difficult/unsolvable task under time pressure and transfer to everyday situations<br><br>*Written self-reflection*<br>Alternatives to habitual stimulus-response patterns;<br><br>*Everyday tasks:*<br>Audio "Open monitoring";<br>Reflection of emotions throughout the day;<br>Attention to habitual self-verbalization;<br>Identification of (habitual) stimulus-response patterns;<br>Exchange with the buddy
Module 5: Social Relationships and Social Behavior	
**Content**	**Exercises/Reflection**
Conclusion of the first topic "Dealing with stress"<br>Reflection on change goals and developmental process<br><br>*Psychoeducation on social relationships and socially competent behavior:*<br>Characteristics of social relationships;<br>Definition of social learning;<br>Common aims of social interactions;<br>Basics of communication;	*Arrival with audio "Open Monitoring" & dyadic/ group exchange*<br><br>*Written self-reflection and interim assessment:*<br>Comparison of past, current, and desired state;<br>Changes in dealing with stress;<br>Use of strengths and resources;<br>Goal adjustments and next steps<br><br>*"Attentive dialog" exercise on change goals:*<br>Attentive listening and feeling;<br>Reflecting on the current situation in social relationships;

Constructivism: reality construction, interdependence, circularity and feedback loops<br><br>*Introduction to the three types of relationship situations:*<br>a) taking responsibility and setting healthy boundaries<br>b) getting into contact/ relationships<br>c) staying in relationships/resonance;<br><br>*Repetition: How to approach sustainable change*	Incorporating resources, needs, and values in social relationships<br><br>*Written self-reflection:*<br>Analysis of the current and the desired state in dealing with social relationships;<br>Analysis of resources and obstacles for change;<br>Commitment to change goal(s)<br><br>*Everyday tasks:*<br>Audio "Open monitoring";<br>Interview "Perception of self and others in social relationships": Interviewing a trusted person on the external perception of the participant in social situations;<br>Exchange with the buddy
Module 6 and Module 7: Practical Exercises I and II	
**Content**	**Exercises/Reflection**
Practical training module:<br>Explanation and introduction of videotaped fictive role plays for different social situations;<br>Specification of the three types of relationship situations and role-plays selecting a situation from one of the types:<br>a) taking responsibility and setting healthy boundaries<br>b) getting into contact/ relationships<br>c) staying in relationships/resonance;	*Arrival with audio "Open Monitoring" & dyadic/ group exchange*<br><br>*Role play in two smaller groups and one trainer with video recording and video feedback:*<br>Re-enactment of a challenging everyday social situation;<br>(Positive) Feedback (from self, trainer, sometimes group)<br>Second round with adaptions<br><br>*Everyday tasks:*<br>Audio "Open monitoring";<br>Practice some social situations in "real life" and reflection on these;<br>Exchange with the buddy
Module 8: Review and Outlook	
**Content**	**Exercises/Reflection**
Summary of training and outlook on everyday life in the future:<br>Appreciation of what has been achieved and planning of next steps	*Arrival with audio "Open Monitoring" & dyadic/ group exchange*<br><br>*Self-reflection:*<br>Comparison of past, current, and desired state;<br>Changes in dealing with social relationships;<br>Use of strengths and resources;<br>Goal adjustments and next steps<br><br>*Exchange in the group:*<br>Final reflection;<br>Personal highlights and "AHA moments"

Note. Reprinted from [1]

Supplementary Table S2

Deviations from the Preregistration

Preregistration	Deviation	Reasoning
**Hypotheses**		The hypotheses were rephrased to improve grammatical clarity and consistency as well as avoiding repetitive phrasing
H1a: Over the course of the training, state emotional well-being improves continuously.<br>H1b. Over the course of the training, state social behavior improves. The improvement is more pronounced during the second half of the training, which focuses on social competencies. (Prereg 1)	(…) over the course of the intervention, state emotional stability (H1a) and state extraversion (H1b) would improve continuously. Regarding the latter, we further stated that the improvement would be more pronounced during the second half of the intervention, which focused on social competencies.	
H1a: Participation in the social-emotional competence training leads to increases in the explicit self-concept of emotional stability.<br>H1b: Participation in the social-emotional competence training leads to increases in the explicit self-concept of extraversion. (Prereg 2)	(…) we expected that the intervention would lead to increases in the explicit self-concept of emotional stability (H2a) and extraversion (H2b)	
H2a. Participation in the social-emotional competence training leads to increases in the implicit self-concept of emotional stability.<br>H2b. Participation in the social-emotional competence training leads to increases in the implicit self-concept of extraversion. (Prereg 2)	(…) as well as the implicit self-concepts of both traits (H3a and H3b, respectively)	
H3. Participation in the social-emotional competence training leads to more pronounced increases in explicit trait self-concepts compared to implicit trait self-concepts. (Prereg 2)	(…) we expected more pronounced increases explicit trait self-concepts compared to implicit trait self-concepts (H4)	
H3a: More pronounced changes in state emotional well-being are associated with stronger changes in trait emotional stability.	We hypothesized that more pronounced changes in state emotional well-being would be associated with stronger changes in trait	

Preregistration	Deviation	Reasoning
H3c: More pronounced changes in state social behavior are associated with stronger changes in trait extraversion. (Prereg 1)	emotional stability (H5a) and more pronounced changes in state social behavior with stronger changes in trait extraversion (H5b).	
H2a. Increases in state emotional well-being are more pronounced among younger adults compared to older adults. H2b. Improvement in state social behavior is more pronounced among younger adults compared to older adults. (Prereg 1)	We (…) hypothesized that the increases in state emotional well-being (H6a) and social behavior (H6b) would be more pronounced among younger adults compared to older adults.	
H4a: Increases in explicit self-concepts are more pronounced among younger adults compared to older adults. H4b. Increases in implicit self-concepts are more pronounced among younger adults compared to older adults. (Prereg 2)	(…) we expected (H7a) that increases in explicit self-concepts and implicit self-concepts (H7b) are more pronounced among younger adults compared to older adults.	
H3b: The association between changes in state emotional well-being and trait emotional stability is more pronounced in younger adults compared to older adults. H3d: The association between changes in state social behavior and trait extraversion is more pronounced in younger adults compared to older adults. (Prereg 1)	(…) we also expected that the association between changes in state emotional well-being and trait emotional stability (H8a), and social behavior and extraversion (8b) would be more stronger in younger adults compared to older adults.	
**Variables**		
State social behavior: 6 Items state social behavior; 1 indicator each of social relationship quantity (i.e., frequency) and quality (i.e., mean of pleasantness & closeness)	We did not include indicators of social relationships.	We aimed to maintain a clear and concise focus on the research questions. Further, as we collected for emotional stability also just state indicators it appeared imbalanced to have more indicators for extraversion.
We preregistered the evaluation of the week and compliance with training tasks as control variables.	We did not include them in statistical models but evaluated and reported them descriptively.	Due to the complexity of the models we decided not to add additional variables.
**Sample** We prefined age criteria as:	Because of difficulties in recruiting participants, we expanded the older age group to include	We aimed to increase the sample size due to power considerations.

Preregistration	Deviation	Reasoning
Age ≤ 18 years or outside the intended age ranges of 18-35 years and 55-80 years by more than 2 years	individuals aged 50 years and older, and we included six participants whose ages fell between the predefined ranges for younger adults (18–35 years) and older adults (50+ years).	
**Manipulation of comparisons**		
During weekly assessments when reflecting on their past week, we assigned participants to focus either on comparing themselves with their previous traits (i.e., past-temporal comparison) or with others (social comparison).<br>We preregistered separate hypotheses for the comparisons and how they interact with traits and age.	We did not include analyses on comparison conditions in this manuscript.	As the scope of this manuscript was already complex and covered 15 hypotheses, we did not include this aspect of the study to not overburden readers.
**Analyses**		
For linking state and trait changes we preregistered that if the data quality is sufficient, we use structural equation models that link changes in weekly experiences (i.e., slopes) with trait changes (i.e., latent neighbor change models).	We used a somewhat simpler model with one latent growth factor.	Models with one growth factor seemed reasonable as the change was equal across both study parts.
We preregistered using MLR estimation.	We used Bayesian estimation.	Neighborhood change models did not converge.

Supplementary Table S3

Dropout and Attrition Analyses

Variables *M(SD)*	Completers (N = 165)[a]	Drop-out (N = 38)[a]	Welch's $t(df)$ or $\chi^2(df)$[b]	p	Adherence[c]	Attrition[c]	Welch's $t(df)$ or $\chi^2(df)$[d]	p
**Explicit**								
Emotional Stability	2.86 (0.60)	2.96 (0.58)	0.94 (51.02)	.352	2.75(0.63)	2.74 (0.51)	-0.11 (19.29)	.916
Extraversion	3.21 (0.64)	3.33 (0.55)	1.26 (55.93)	.213	3.40 (0.61)	3.46 (0.42)	-0.53 (21.66)	.599
**Implicit**								
Emotional Stability	0.33 (0.37)	0.29 (0.29)	-0.50 (48.93)	.618	0.30 (0.41)	0.11 (0.36)	1.77 (17.07)	.095
Extraversion	-0.21 (0.56)	-0.15 (0.54)	0.47 (44.10)	.638	-0.10 (0.56)	0.11 (0.55)	-1.32 (16.19)	.207
Age	46.26 (18.70)	47.81 (18.60)	0.46 (53.58)	.649	45.38 (18.32)	49.32 (19.93)	1.08 (54.80)	.285
Gender (*n*)			1.45 (2)	.484			1.03 (2)	.597
Female	124	24			98	26		
Male	40	12			29	11		
Non-binary	1	0			1	0		

Note. 36.3% of the eligible participants (N = 203) eventually enrolled in the study. Of these, 18.7% (n = 38) decided not to start (n = 20) or to end participation prematurely (i.e., did only complete ≤ 4 training sessions; n = 18). These are considered a drop-out. [a]Data is available from $n_{\text{Completers}}$ = 158-165; $n_{\text{Drop-out}}$ = 30-37 at T1. [b]Mean difference at T1 between completers and drop-out. [c]Adherence = Participants provided data at T4 and/or T5 (n = 124-128). Attrition = Participants did not provide data at either follow-up (n = 14-37). [d]Mean difference at T3 between adherence and attrition.

Supplementary Material

Supplementary Figure S1

Study Flow and Attrition

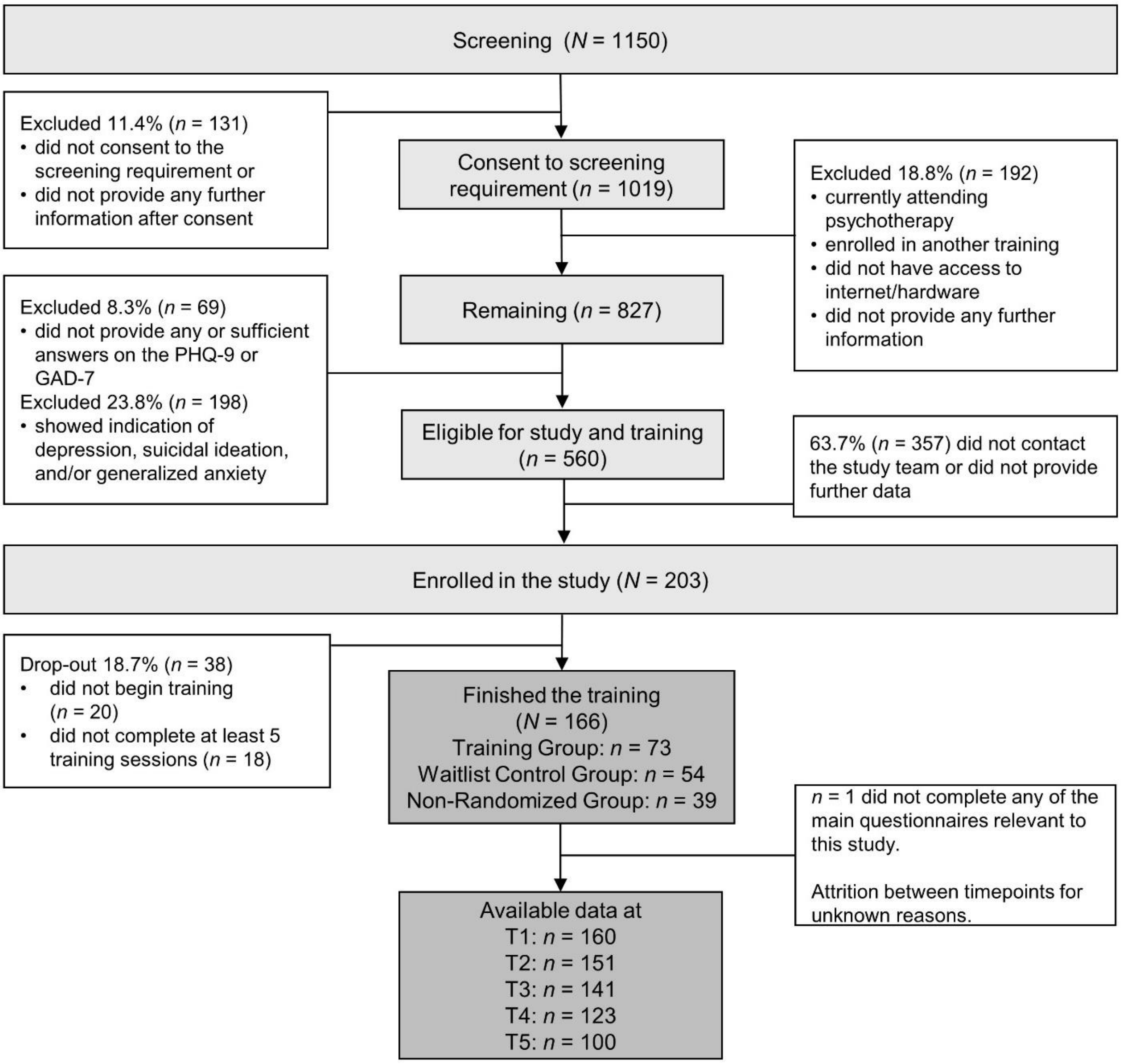

Note. Reprinted from[1]. The figure depicts the study flow and attrition. Some individuals may have repeatedly participated in the screening. More than one exclusion criterion may apply to one individual. Not all participants who completed the training filled out all study questionnaires at all time points, therefore the *n* varies across time points.

Supplementary Table S4

Sociodemographic Information on Participants

Variable	Overall (*N* = 165)	Younger adults (*n* = 80)	Older adults (*n* = 85)
**Age**			
Mean (SD)	46.26 (18.70)	28.33 (4.92)	63.55 (7.20)
Range	19-78	19-42	50-78
**Gender *n* (%)**			
Female	124 (75.15)	61 (75.31)	63 (75.00)
Male	40 (24.24)	19 (23.46)	21 (25.00)
Non-Binary	1 (0.61)	1 (1.23)	0 (0.00)
**Education *n* (%)**[a]			
Up to middle school degree	14 (8.48)	2 (2.47)	12 (14.29)
High school degree	29 (16.97)	21 (25.93)	8 (9.52)
College degree (UAS)	5 (3.03)	1 (1.23)	4 (4.76)
University degree	104 (63.03)	53 (65.43)	51 (60.71)
Other	9 (5.45)	4 (4.94)	5 (5.95)
**Job *n* (%)**[a]			
Student	45 (27.27)	45 (55.56)	0 (0.00)
Employee	65 (39.39)	30 (37.04)	35 (41.67)
Self-Employed	4 (2.42)	0 (0.00)	4 (4.76)
Pensioner	36 (21.82)	0 (0.00)	36 (42.86)
Other	11 (6.67)	6 (7.41)	5 (5.95)
**Civil Status *n* (%)**[a]			
Married	48 (29.09)	9 (11.11)	39 (46.43)
In a relationship	41 (24.85)	36 (44.44)	5 (5.95)
Single	47 (28.48)	35 (43.21)	12 (14.29)
Divorced	19 (11.52)	1 (1.23)	18 (21.43)
Widowed	6 (3.64)	0 (0.00)	6 (7.14)
**Kids *n* (%)**[a]			
None	97 (58.79)	76 (93.83)	21 (25.00)
1	17 (10.30)	3 (3.70)	14 (16.67)
2	35 (21.21)	1 (1.23)	34 (40.48)
3	9 (5.45)	1 (1.23)	8 (9.52)
4	3 (1.82)	0 (0.00)	3 (3.54)
**Net Household Income in € *n* (%)**[a]			
< 1.000	41 (24.85)	33 (40.74)	8 (9.82)
1.000 - 2.000	31 (18.79)	14 (17.28)	17 (20.24)
2.000 - 3.000	42 (25.45)	18 (22.22)	24 (28.57)
3.000 - 5.000	29 (17.58)	10 (12.35)	19 (22.62)
5.000 - 7.500	6 (3.64)	0 (0.000)	6 (7.14)
7.500 - 10.000	2 (1.21)	0 (0.00)	2 (2.38)
> 10.000	1 (0.61)	0 (0.00)	1 (1.19)
Not indicated	9 (5.45)	6 (7.41)	3 (3.57)

Note. UAS = University of Applied Sciences

Supplementary Table S5

Correlations Between Explicit and Implicit Self-Concepts at all Measuring Points

	1	2	3	4	5	6	7	8	9	10	11	12	13	14	15	16	17	18
1. ES explicit T1																		
2. ES explicit T2	**.79**																	
3. ES explicit T3	**.77**	**.84**																
4. ES explicit T4	**.73**	**.79**	**.82**															
5. ES explicit T5	**.52**	**.58**	**.66**	**.75**														
6. ES implicit T1	.08	.21	.15	.10	-.07													
7. ES implicit T2	**.25**	**.31**	.22	.19	.10	**.48**												
8. ES implicit T3	.20	**.23**	.16	.16	.17	**.36**	**.49**											
9. ES implicit T5	.08	.24	.12	.04	.06	**.28**	**.46**	**.32**										
10. EX explicit T1	**.27**	**.25**	.18	**.29**	.15	.07	.14	**.25**	.09									
11. EX explicit T2	**.22**	**.28**	**.23**	**.28**	**.28**	.09	.18	.22	.09	**.87**								
12. EX explicit T3	**.23**	**.27**	**.28**	**.31**	**.28**	.07	.22	**.23**	.07	**.86**	**.92**							
13. EX explicit T4	**.30**	**.35**	**.32**	**.44**	**.33**	.09	.18	.17	.00	**.85**	**.87**	**.88**						
14. EX explicit T5	.23	.20	.24	**.37**	**.47**	-.06	.12	.18	.02	**.76**	**.81**	**.81**	**.83**					
15. EX implicit T1	**.25**	.17	.21	.24	.17	-.01	.00	.12	-.16	**.33**	**.43**	**.41**	**.45**	**.36**				
16. EX implicit T2	.14	.09	.06	.17	.01	.07	.07	.05	-.21	**.32**	**.36**	**.32**	**.43**	**.31**	**.64**			
17. EX implicit T3	.05	.04	.11	.16	.09	.16	.10	.00	-.13	**.31**	**.39**	**.34**	**.46**	**.31**	**.57**	**.67**		
18. EX implicit T5	.12	.17	.23	.22	.17	.10	.09	-.03	.01	.23	**.29**	**.29**	**.30**	.23	**.45**	**.50**	**.64**	
19. Age	-.01	-.02	.05	.07	.08	.06	.05	.04	.25	.00	-.04	-.05	-.11	-.01	.02	-.06	-.05	.00

Note. ES = Emotional stability, EX = Extraversion. Significant correlations are bolded with $p < .05$.

Supplementary Table S6

Fit Indices for Models with Different Levels of Measurement Invariance Across the Intervention

Model	χ^2	CFI	TLI	RMSEA	SRMR
**ES explicit**					
1	6.549	1.000	1.018	0.000	0.023
2	10.317	1.000	1.015	0.000	0.025
3	32.983	0.998	1.010	0.051	0.053
**EX explicit**					
1	11.894	1.000	1.006	0.000	0.018
2	15.774	1.000	1.005	0.000	0.026
3	16.277	1.000	1.008	0.000	0.028
**ES implicit**					
1	0.826	0.997	1.000	0.000	0.009
2	2.160	0.999	0.996	0.022	0.024
3	3.329	1.000	1.009	0.000	0.028
**EX implicit**					
1	0.385	0.999	1.000	0.000	0.004
2	1.892	1.000	1.001	0.000	0.020
3	1.927	1.000	1.013	0.000	0.020

Note. ES = Emotional stability, EX = Extraversion. CFI = Comparative fit index; TLI = Tucker-Lewis index; RMSEA = Root mean square error of approximation; SRMR = Standardized root mean square residual. 1 = Unconstrained model/configural measurement invariance; 2 = Model with metric measurement invariance; 3 = Model with strong measurement invariance. For testing Measurement Invariance, we used the following guidelines of Chen (2007)[2] for samples with $N \leq 300$: To test configural invariance: Change of ≤—.005 in CFI, supplemented by a change of ≥.010 in RMSEA or a change of ≥.025 in RMSEA compared to the less restrictive model would indicate noninvariance: Metric invariance and strong invariance: change of ≥ —.005 in CFI, supplemented by a change of ≥.015 in RMSEA or a change of ≥.010 in SRMR compared to the less restrictive model would indicate noninvariance

Supplementary Table S7

Model Fit Indices of Latent Difference Models (Model A)

Model	χ^2	CFI	TLI	RMSEA	SRMR
A: Latent neighbor change					
ES explicit	43.985	0.992	0.990	0.037	0.052
EX explicit	37.642	0.999	0.998	0.017	0.070
ES implicit	6.666	1.000	1.000	0.000	0.031
EX implicit	5.199	1.000	1.000	0.000	0.018

Note. ES = Emotional stability, EX = Extraversion. CFI = Comparative fit index; TLI = Tucker-Lewis index; RMSEA = Root mean square error of approximation; SRMR = Standardized root mean square residual.

Supplementary Table S8

Model Fit Indices of Latent Growth Models with Bayes Estimator (Model B)

**Model**	CFI	TLI	RMSEA	BIC
ES explicit	0.971	0.971	0.042	5573.280
EX explicit	1.000	1.000	0.000	4244.147
ES implicit	0.992	0.992	0.025	4966.628
EX implicit	1.000	1.000	0.000	4001.943

Note. ES = Emotional stability, EX = Extraversion. CFI = Comparative fit index; TLI = Tucker-Lewis index; RMSEA = Root mean square error of approximation; BIC = Bayesian information criteria.

Supplementary Table S9

Model Fit Indices of Follow-Up Analyses (Model C)

**Model**	CFI	TLI	RMSEA	BIC
ES explicit	1.000	1.000	0.000	2772.628
EX explicit	0.940	0.938	0.082	2519.236
ES implicit	1.000	1.000	0.000	739.120
EX implicit	1.000	1.000	0.000	1069.906

Note. ES = Emotional stability, EX = Extraversion. CFI = Comparative fit index; TLI = Tucker-Lewis index; RMSEA = Root mean square error of approximation; BIC = Bayesian information criteria.

Supplementary Table S10

Changes in Personality States Across the Intervention

**Fixed Effect**	**State Emotional Stability**	**State Extraversion**
**Intercept**		
Estimate (SE)	**4.11 (0.08)**	**4.70 (0.08)**
95% CI	[3.95, 4.27]	[4.55, 4.85]
**Time**		
Estimate (SE)	**0.09 (0.02)**	**0.06 (0.01)**
95% CI	[0.06, 0.13]	[0.03, 0.08]
**Age Group**[a]		
Estimate (SE)	0.19 (0.19)	0.20 (0.16)
95% CI	[-0.13, 0.50]	[-0.11, 0.50]
**Time by Age Group**		
Estimate (SE)	-0.01 (0.03)	-0.01 (0.02)
95% CI	[-0.08, 0.05]	[-0.06, 0.03]

Note. [a] 0 = younger adults, 1 = older adults, grand-mean centered. Coefficient with *p*-values <.05 are bolded. *N* =1176.

Supplementary Table S11

Changes in Social Personality States Across the Intervention with a Discontinuous Time Variable

**Fixed Effect**	**State Social Behavior**
**Intercept**	
Estimate (SE)	**4.72 (0.08)**
95% CI	[4.55, 4.88]
**Time**	
Estimate (SE)	0.04 (0.03)
95% CI	[-0.01, 0.10]
**Time Discontinuous**[a]	
Estimate (SE)	0.03 (0.02)
95% CI	[-0.30, 0.35]
**Age Group**[b]	
Estimate (SE)	0.14 (0.13)
95% CI	[-0.10, 0.39]
**Time by Time Discontinuous**	
Estimate (SE)	0.01 (0.04)
95% CI	[-0.07, 0.09]

Note. *N* =1176. 0 = younger adults, 1 = older adults, grand-mean centered.
Coefficient with *p*-values < .05 are bolded.

Supplementary Table S12

Longitudinal Change in Explicit and Implicit Self-Concepts of Emotional Stability and Extraversion (Model A)

Model	Emotional stability		Extraversion	
	Estimate [95% CI]	*p*	Estimate [95% CI]	*p*
**Explicit**				
Trait changes				
Change T1–T2	0.321 [0.229, 0.412]	**<.001**	0.161 [0.098, 0.225]	**<.001**
Change T2–T3	0.319 [0.222, 0.416]	**<.001**	0.072 [0.004, 0.140]	**.037**
Neighborhood change T1–T2→ T2–T3	-0.398 [-0.621,-0.174]	**<.001**	-0.088 [-0.380, 0.203]	.552
Age differences in change				
Change T1–T2	0.042 [-0.109, 0.192]	.586	-0.004 [-0.123, 0.116]	.953
Change T2–T3	0.092 [-0.037, 0.221]	.162	0.049 [-0.056, 0.154]	.359
**Implicit**				
Trait changes				
Change T1–T2	0.002 [-0.059, 0.063]	.944	0.098 [0.024, 0.172]	**.009**
Change T2–T3	-0.049 [-0.105, 0.006]	.082	0.062 [0.000, 0.124]	.051
Neighborhood change T2–T1 → T2–T3	-0.025 [-0.047, -0.004]	**.019**	-0.324 [-0.570, -0.077]	**.010**
Age differences in change				
Change T1–T2	0.033 [-0.068,0.134]	.520	-0.090 [-0.220, 0.040]	.174
Change T2–T3	-0.008 [-0.116, 0.100]	.887	0.024 [-0.083, 0.132]	.657

Note. $N = 165$. Coefficient with *p*-values < .05 are bolded.

Supplementary Table S13

State-Trait Associations and Moderations by Age in Longitudinal Change in the Explicit and Implicit Self-Concepts of Emotional Stability and Extraversion (Model B)

Model	Emotional stability			Extraversion		
	Estimate [95% CI]	*Posterior SD*	*p*	Estimate [95% CI]	*Posterior SD*	*p*
**Explicit**						
a Association of intercepts	0.237 [0.143, 0.356]	0.054	**<.001**	0.364 [0.238, 0.524]	0.073	**<.001**
b State changes predict trait changes	0.367 [0.191, 0.674]	0.132	**<.001**	0.010 [-0.239, 0.290]	0.135	.458
c State changes moderated by age group predict trait changes	-0.148 [-0.414, 0.092]	0.128	.111	-0.029 [-0.392, 0.322]	0.184	.431
**Implicit**						
a Association of Intercepts	0.015 [-0.039, 0.070]	0.027	.289	**0.091 [0.020, 0.175]**	**0.039**	**.006**
b State changes predict trait changes	0.018 [-0.059, 0.117]	0.044	.317	0.010 [-0.942, 0.299]	0.291	.465
c State changes moderated by age group predict trait changes	0.039 [-0.139, 0.224]	0.092	.322	-0.116 [-2.033, 0.450]	0.571	.294

Note. N = 165. Coefficient with p-values < .05 are bolded.

Supplementary Table S14

BIC Differences of Latent Growth Models With and Without Age Group as Predictor

Model	BIC H1 (with)	BIC H0 (without)	BIC Difference	Evidence against H1 (Bayes factor)
State-Trait Associations across the Intervention (Model B)				
ES explicit	5573.280	5569.990	3.290	
EX explicit	4966.628	4962.019	4.609	Positive evidence for H0 (3 to 20)
ES implicit	4244.147	4238.938	5.209	
EX implicit	4001.943	3997.744	4.199	
Trait Changes Across the Intervention and Follow-Up Period (Model C)				
ES explicit	2772.628	2765.595	7.033	Strong evidence for H0 (20 to 150)
EX explicit	2515.943	2519.518	3.575	Positive evidence for H0 (3 to 20)
ES implicit	739.120	734.088	5.032	
EX implicit	1069.906	1063.747	-6.159	Strong evidence for H1 (20 to 150)

Supplementary Table S15

Longitudinal Change in Explicit and Implicit Self-Concepts of Emotional Stability and Extraversion Across the Intervention and Follow-Up Period (Model C)

Model	Emotional stability			Extraversion		
	Estimate [95% CI]	*Posterior SD*	*p*	Estimate [95% CI]	*Posterior SD*	*p*
**Explicit**						
Trait changes across the intervention	0.265 [0.221, 0.308]	0.022	**<.001**	0.100 [0.060, 0.140]	0.020	**<.001**
Trait changes across follow-up period	0.004 [-0.006, 0.014]	0.005	.201	-0.010 [-0.017, -0.003]	0.003	**.002**
Age group predicting changes across the follow-up period	0.004 [-0.014, 0.023]	0.009	.327	-0.002 [-0.015, 0.010]	0.006	.384
**Implicit**						
Trait changes across the intervention	-0.024 [-0.058, 0.009]	0.017	.075	0.065 [0.026, 0.104]	0.020	**<.001**
Trait changes across the follow-up period	0.005 [-0.002, 0.011]	0.003	.069	0.006 [-0.002, 0.013]	0.004	.064
Age group predicting changes across the follow-up period	0.010 [-0.004, 0.022]	0.006	.071	0.016 [0.001, 0.031]	0.008	**.017**

Note. *N(T4) = 123, n(T5) = 100*. Age group (0 = younger adults, 1 = older adults) was grand mean centered. Coefficient with *p*-values < .05 are bolded.

Supplementary Table S16

Age Differences in the Engagement in the Intervention and Context Factors

Variables M(SD)	Younger Adults	Older Adults	*t (df)*	*p*
**Desire to Change**				
Emotional Stability	4.13 (0.56)	4.02 (0.59)	1.29 (158.78)	.199
Extraversion	3.60 (0.55)	3.40 (0.56)	2.24 (159)	**.026**
**Engagement**				
Weekly tasks	3.49 (1.14)	4.31 (1.00)	-4.89 (155.15)	**< .001**
Practicing with audio files	3.08 (1.40)	3.89 (1.44)	-3.64 (159.90)	**< .001**
Exchanging with Buddy	2.13 (0.61)	2.23 (0.73)	-0.95 (157.28)	.340
Practicing in daily live	4.10 (1.10)	4.38 (0.99)	-1.60 (156.19)	.110
**Context factors**				
Hectic Week	4.48 (0.89)	4.11 (0.99)	2.49 (159.49)	**.013**
Exhausting Week	4.31 (0.73)	4.32 (0.63)	-0.10 (153.60)	.921
Atypical Week	4.33 (0.96)	3.80 (1.13)	3.23 (158.04)	**.002**

Note. Significant *p*-values < .05 are bolded.

Supplementary Table S17

Results of Monte Carlo Simulations Comparing the Planned and Final Sample Sizes

Parameters	% models with estimate within 95% CI	% models with sign. estimate
Extraversion, *N* = 165		
age effects in intercept b = -.01	96%	4%
age effects in slope b = .01	94%	20%
Extraversion, *N* = 220		
age effects in intercept b = -.01	95%	5%
age effects in slope b = .01	94%	22%
Emotional stability, *N* = 165		
age effects in intercept b = .02	95%	6%
age effects in slope b = .07	95%	100%
Emotional stability, *N* = 220		
age effects in intercept b = .02	95%	5%
age effects in slope b = .07	94%	100%

Note. We always used 1000 replications.

Supplemental References

1. Borgdorf, K. S. A., Küchler, G., Wrzus, C. & Aguilar-Raab, C. Mindful and well: The effects of a socioemotional competence training (SECT) in a randomized controlled trial. *J. Couns. Psychol.* **72**, 329–341 (2025).
2. Chen, F. F. Sensitivity of Goodness of Fit Indexes to Lack of Measurement Invariance. *Struct. Equ. Model. Multidiscip. J.* **14**, 464–504 (2007).
